# Performance of distributed multiscale simulations

**DOI:** 10.1098/rsta.2013.0407

**Published:** 2014-08-06

**Authors:** J. Borgdorff, M. Ben Belgacem, C. Bona-Casas, L. Fazendeiro, D. Groen, O. Hoenen, A. Mizeranschi, J. L. Suter, D. Coster, P. V. Coveney, W. Dubitzky, A. G. Hoekstra, P. Strand, B. Chopard

**Affiliations:** 1 Computational Science, Informatics Institute, University of Amsterdam, Science Park 904, 1098 XH Amsterdam, The Netherlands; 2 Computer Science Department, University of Geneva, 1227 Carouge, Switzerland; 3 Department of Applied Mathematics, University of A Coruña, 15001 A Coruña, Spain; 4 Department of Earth and Space Sciences, Chalmers University of Technology, 41296 Göteborg, Sweden; 5 Centre for Computational Science, University College London, 20 Gordon Street, London WC1H OAJ, UK; 6 Max-Planck-Institut für Plasmaphysik, 85748 Garching, Germany; 7 Nano Systems Biology, School of Biomedicine, University of Ulster, Coleraine BTS2 1SA, UK; 8 National Research University ITMO, Kronverkskiy prospekt 49, 197101 St Petersburg, Russia

**Keywords:** distributed multiscale computing, performance, multiscale simulation

## Abstract

Multiscale simulations model phenomena across natural scales using monolithic or component-based code, running on local or distributed resources. In this work, we investigate the performance of distributed multiscale computing of component-based models, guided by six multiscale applications with different characteristics and from several disciplines. Three modes of distributed multiscale computing are identified: supplementing local dependencies with large-scale resources, load distribution over multiple resources, and load balancing of small- and large-scale resources. We find that the first mode has the apparent benefit of increasing simulation speed, and the second mode can increase simulation speed if local resources are limited. Depending on resource reservation and model coupling topology, the third mode may result in a reduction of resource consumption.

## 1. Introduction

Multiscale modelling and simulation is a field receiving wide interest [1], from mathematics [2], biology [3–5], physics [6–9], engineering [10,11] and many other disciplines. A small number of conceptual frameworks provide an over-arching view of multiscale modelling [6,12,13]; some of these take a scale-aware component-based modelling approach.

This work adopts one such approach, the Multiscale Modelling and Simulation Framework (MMSF) [13] (see also the review by Chopard *et al.* in this Theme Issue [14]), which defines a multiscale model as a set of coupled single-scale models. The framework gives guidelines and tools for constructing, describing and implementing multiscale models in a component-based way. Its aim is to be able to provide general software to simulate these multiscale models, by standardizing their coupling and communication aspects. The framework is based on the concept of complex automata, which couples cellular automata of different scales together [15,16]. The framework distinguishes between cyclic and acyclic coupling topologies, dependent on the presence or the absence of feedback loops. It allows for tight interactions, in contrast with many scientific workflow paradigms [17,18].

Over the past few years, we have developed a large collection of multiscale models [19–24] and have found that these multiscale models are computationally intensive. Other examples of such demanding multiscale models are Earth system models [9,25–27], each taking a component-based approach with the possibility for distributed computing. These models can be executed on a single cluster or supercomputer; however, when considering multiple coupled submodels, a single resource may not be suitable or sufficient to run all submodels. This may be because the submodels have different (licensed) software dependencies, need specific hardware such as general-purpose computing on graphics processing units (GPGPUs), fast input/output (I/O) or a very large number of processors to compute efficiently, or need access to a local database. Even a single submodel may need more processors than are available on any one cluster. On the other hand, to simply run all submodels on a high-performance computing (HPC) resource that provides for all needs is not always possible and certainly not always efficient, since the submodels may have highly heterogeneous characteristics. At the high end of computing, even exascale simulations will likely feature significant heterogeneity in I/O and CPU requirements [28]. In a component-based approach, submodel code may be replaced to match a given architecture without changing other parts of the model, or submodels may be distributed over the resources that fit their needs. The former approach may be desirable, but the latter is less invasive to the code and the model, and, depending on the communication overhead, may be beneficial for efficiency.

This work analyses the advantages of the component-based approach and assesses the overhead involved in doing distributed multiscale computing. This is motivated by the recently completed MAPPER project,^1^ which aimed to facilitate large multiscale simulations on distributed e-Infrastructure. The project was driven by seven multiscale applications from the following disciplines: nano materials [22], fusion [21], biomedicine [23], hydrology [29] and systems biology [20]. We divide these applications into three categories based on how they may benefit from distributed computing:

(i) by increasing simulation speed by supplementing local dependencies (e.g. specific software or hardware) with large resources (e.g. supercomputers);
(ii) by increasing simulation speed through using more resources than available to a single computer or cluster; and
(iii) by increasing resource efficiency through running each submodel on appropriate computing resources.

In MAPPER, we have chosen MUSCLE 2 [30] and MPWide [31] as coupling technologies for cyclic models, where submodels must communicate frequently, and the GridSpace Experiment Workbench (EW) [32,33] for acyclic coupling topologies. These technologies have local and distributed computing capabilities. Applications with homogeneous code or a high ratio of communication over computation, or situations where the researcher has a very flexible local resource available, will likely be more suitable for local computing and were not present in the project.

## 2. Multiscale modelling and simulation framework

We define multiscale models as coupled single-scale models [13] and characterize coupling topologies as cyclic or acyclic. A cyclic coupling topology involves feedback between single-scale models, whereas acyclic coupling topologies do not. Moreover, pairs of interacting single-scale models are characterized by having either temporal scale separation or overlap. According to MMSF coupling templates, submodels with temporal scale overlap exchange messages during their execution and are able to run in parallel. Indeed, they may need to run concurrently to be able to exchange data. By contrast, submodels whose time scales are separated run sequentially, so they will generally not be able to compute in parallel.

In MAPPER, we have defined a tool chain [33] to compute multiscale models that can be described with the MMSF. It starts by specifying the architecture with the Multiscale Modelling Language (MML) [13] in a dedicated user interface and then executing it with the GridSpace Experiment Workbench [32] for acyclic coupling topologies, and MUSCLE 2 [30], if needed in combination with MPWide [31], for cyclic coupling topologies. Distributed multiscale simulations are coordinated by middleware, in our case QCG-Broker [34] and the Application Hosting Environment [35]. Zasada *et al.* [36] describe the MAPPER infrastructure in more detail. Middleware is likely to play an important role to ease the transition to distributed computing by managing the resources from a central location and arranging co-allocated resources.

## 3. Performance context

When is distributed multiscale computing a valuable addition to multiscale modelling and simulation? We identify three key aspects to this question: how will the understanding and development time of a multiscale model benefit from modularization; how long does it take to complete a simulation; and how many resources are used in the process. Ideally, the development time, the time to complete a simulation (makespan) and the amount of required resources are minimized. In practice, these aspects have to be balanced, so as not to increase resources usage exorbitantly for a small gain in performance or to sacrifice performance for the sake of the lowest resource usage.

Already when modelling, a multiscale model may benefit from modularization by dissecting it into multiple coupled single-scale models, because this also signifies a separation of concerns common in component-based software design [37,38]. Each submodel in a multiscale model should be independently correct, which will in some cases be easier to validate than validating an entire monolithic model at once. Better yet, a well-validated model may already exist for part of the multiscale model. Separating the multiscale model into single-scale submodels also makes it easier to replace part of the model if, for example, more detail or a faster solving method is needed. However, it may be very hard, both theoretically and computationally, to separate a model into multiple parts if these are intrinsically and closely linked. For example, two submodels that need to exchange large quantities of data every few milliseconds may benefit from faster communication methods by putting them in a single submodel and code.

**Figure 1.**
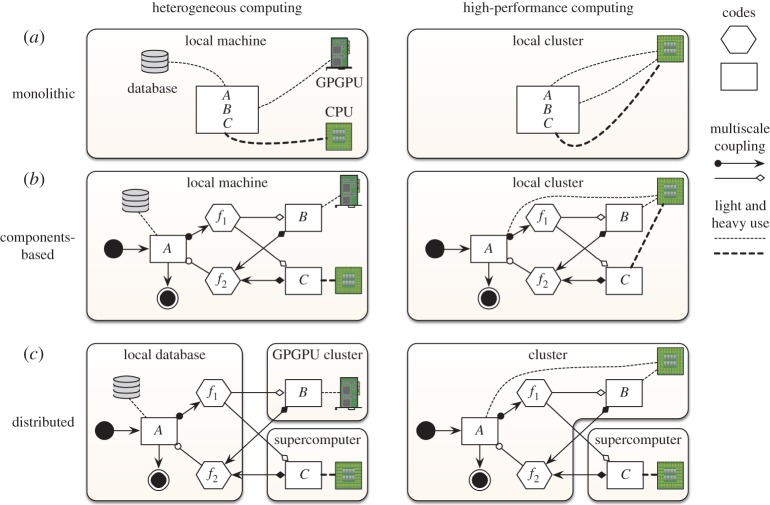
Scenarios using component-based modelling or distributed computing. (*a*) A monolithic model incorporating all codes *A*, *B*, *C* into a single code base. (*b*) The model is decomposed into submodels and the codes are separated by function, also separating the runtime dependencies per submodel. (*c*) How the components could be distributed to increase resource effectiveness. (Online version in colour.)

Regarding the implementation of a multiscale model, having parts of the model available as separate submodels makes it possible to apply techniques that are most useful for one submodel but not another, as outlined in figure 1. Thus, it is possible to implement a multiscale model by combining several programming languages (an existing Fortran code with a C++ library) or techniques (GPU computing with scalable Message Passing Interface (MPI) and OpenMP). During execution of the multiscale model, submodels should ideally run on the hardware that is best suited for them, for example, scalable MPI code on a supercomputer and GPU code on a GPGPU cluster, and in a suitable environment, with the required software site licences and software dependencies. All these preconditions might not be satisfied on a single machine while they may be on a (distributed) set of machines. While combining codes may help modelling and code reuse, the communication between submodels should not become a bottleneck.

Applications can be grouped based on what advantage distributed computing has for them. In the first category, *tied* multiscale simulations have at least one submodel tied to a certain machine, and by using distributed computing other submodels are no longer tied to that machine so they can run more efficiently elsewhere. In the second category, *scalable* multiscale simulations can take advantage of using more machines to run simulations faster or with a larger problem size. In the third category, *skewed* multiscale simulations may run on supercomputers but they consume fewer resources by running less demanding submodels on machines with fewer cores.

Consider a multiscale model as a set of coupled submodels *s*_1_,…,*s*_*n*_. The time to compute a submodel depends on the architecture of the resource it runs on, and the number of cores that it uses on that resource. A submodel *s*_*i*_ may run on architecture *a*_*j*_∈*A*(*s*_*i*_), where *A*(*s*_*i*_) denotes the set of admissible architectures for *s*_*i*_. The time to compute submodel *s*_*i*_ on *a*_*j*_ with *p* cores is then *t*_*i*_(*a*_*j*_,*p*). We assume that the local communication time *c*_local_ is less than the distributed communication time *c*_distr_. The makespan (total time a model takes to completion) on local resources is *T*_local_, using *R*_local_ CPU hours;^2^ the makespan on distributed resources is *T*_distr_, using *R*_distr_ CPU hours. The speed-up *Sp* and relative resource use *U* of distributed computing are defined as

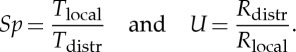



For simplicity, the performance models are reduced to submodels *s*_*i*_ and architectures *a*_*i*_ with *i*=1,2. Much more detail is possible for each of the applications individually, and this will be reported elsewhere. For our current purposes, considering two submodels on two architectures is sufficient.

## 4. Results

The multiscale applications in this study are divided into three groups based on the benefits they derive from distributed multiscale computing, as mentioned in the introduction. The multiscale models consist of the following:

*Tied multiscale models.* A tokamak plasma model (Transport Turbulence Equilibrium, TTE) from the fusion community [21] and a cerebrovascular blood flow model (HemeLB) from the biomedical community [23].
*Scalable multiscale models.* A model to reverse-engineer gene-regulatory networks (MultiGrain) from the systems biology community [20] and an irrigation network model (Canals) from the hydrology community [29].
*Skewed multiscale models.* A model of in-stent restenosis (ISR3D) from the biomedical community [23,24] and a clay–polymer nanocomposites model (Nano) from the nanomaterial community [22,39].

The details of these models can be found in appendix A. Detailed measurements can be found in the electronic supplementary material.

### (a) Tied multiscale computing

The TTE application depends on a local database and HemeLB on specific Python modules, forcing the use of low-performance computing resources to execute at least part of the computations. Examples dealing with similar restrictions include the need for specific hardware or a software site licence. By using distributed multiscale computing, small resources are still used to satisfy these dependencies, but they can be supplemented with larger resources where possible to decrease the simulation time.

For tied multiscale models, consider the following model: *A*(*s*_1_)={*a*_1_}, *A*(*s*_2_)={*a*_1_,*a*_2_} and *t*_2_(*a*_1_,*p*_1_)>*t*_2_(*a*_2_,*p*_2_), where *p*_*i*_ is the number of used cores on *a*_*i*_. Locally, on *a*_1_, the makespan would be

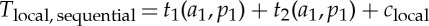

for two sequentially executing submodels and

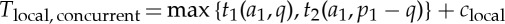

for two concurrently executing submodels, where 1≤*q*<*p*_1_ cores are assigned to one submodel, and the remaining cores to the other submodel. The resource used would be *R*_local, mode_=*p*_1_*T*_local, mode_. In a distributed setting, that would be
4.1
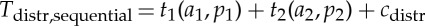

and
4.2
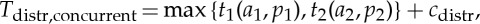

with *R*_distr, mode_=(*p*_1_+*p*_2_)*T*_distr, mode_. For sequentially executing submodels, distributed multiscale computing will yield a shorter makespan if

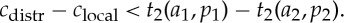

The makespan is shorter for concurrently executing submodels, if

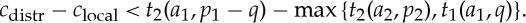

In both cases, the resource usage may increase since usually *p*_1_*t*_2_(*a*_1_,*p*_1_)<*p*_2_*t*_2_(*a*_2_,*p*_2_), which may be acceptable if the decrease in makespan is significant.

The performance for TTE and HemeLB is listed in table 1. The TTE application needs to use a local database on the Gateway cluster (table 2) from which experimental and simulation data are accessed through an application-specific library. At each iteration, a short serial one-dimensional computation is performed on such data before a three-dimensional parallel computation is required. The database is located in the Gateway cluster in Germany with 256 available cores (16 cores per node), but the application also has access to Helios, a community-dedicated supercomputer in Japan (table 2). Per iteration, the serial part takes less than a second on the local cluster, but the parallel part takes over 390 s. If simulations can be distributed between Gateway and Helios, the parallel submodel can scale up to 1024 cores on such use cases, so that the parallel part takes less than 56 s, while increasing the communication time to about 9 s. Despite this increase, the distributed scenario is seven times as fast as the local one.

**Table 1.** Performance measures of tied multiscale models TTE and HemeLB. Owing to the supercomputer policy restricting connections, the distributed communication speed of TTE could not be experimentally verified. Distributed communication time is estimated as *c*_distr_≈5 s, based on network speeds from Germany to Japan (with a latency up to 0.5 s and throughput at least 20 MB *s*^−1^).

simulation	*p*_1_	*T*_local_ (s)	*p*_1_+*p*_2_	*T*_distr_ (s)	speed-up	resources used
TTE	128	397	16+512	98	4.0	1.0
			16+1024	56	7.1	1.2
	256	201	16+512	98	2.0	1.0
			16+1024	56	3.6	1.1
HemeLB	4	144 81	4+512	298	48.6	2.7
			4+2048	157	92.2	5.6

**Table 2.** Resources used for performance measurements in §4. The total number of cores is listed in the right-most column, although practically a fraction of that can be used in a single reservation.

resource	location	type	CPU architecture	cores
Mavrino	London, UK	cluster	Intel Xeon X3353	64
Gordias	Geneva, Switzerland	cluster	Intel Xeon E5530	224
Gateway	Munich, Germany	cluster	Intel Xeon E5-2670	256
Scylla	Geneva, Switzerland	cluster	Intel Xeon Westmere	368
Inula	Poznań, Poland	cluster	AMD Opteron 6234	1600+
Reef	Poznań, Poland	cluster	Intel Xeon E5530	2300+
Zeus	Krakow, Poland	HPC	Intel Xeon L/X/E 56XX	12 000+
Cartesius	Amsterdam, The Netherlands	HPC	Intel Xeon E5-2695 v2	12 500+
Helios	Aomori, Japan	HPC	Intel Xeon E5-2680	70 000+
HECToR	Edinburgh, UK	HPC	AMD Opteron Interlagos	90 000+
SuperMUC	Munich, Germany	HPC	Intel Xeon E5-2680 8C	150 000+

For HemeLB, a local machine with full access is used to install the necessary dependencies for part of the calculations. Since this machine has only four cores, running one iteration of a very well-parallelized code there takes 4 h, whereas pairing the simulation with the HECToR supercomputer reduces the runtime to a few minutes. HemeLB has been shown to scale linearly up to 32 768 cores for simulation domains of approximately 90 M lattice sites [40]. However, here we used a simulation domain of limited size (4.2 M lattice sites). As a result, we observe an increase in resources used for the 512 core run and, especially, for the 2048 core run.

### (b) Scalable multiscale computing

The calculations of MultiGrain revolve around a multi-swarm particle swarm optimization, which as the parameter space gets larger benefits in accuracy and convergence from a larger number of particles grouped in a larger number of swarms. However, Java processes with file-based communication were used for the simulations, essentially limiting the computations to a single machine. This set-up is still possible using MUSCLE 2, but if needed distributed computing can be used to involve more nodes in the computation to scale it up. For the Canals application, although the canal sections in an irrigation network are simulated with fully parallelized code, a supercomputer or multiple clusters are necessary to simulate realistic irrigation network topologies within acceptable timespans. By using distributed multiscale computing, the total number of compute nodes may scale with the size of the network, or a single canal section may be solved faster to provide real-time feedback to a user.

Scalable multiscale models can be modelled with *A*(*s*_1_)=*A*(*s*_2_)={*a*_1_,*a*_2_}, with *p* cores used on both sites, and can be approached with a weak or strong scaling approach: scaling the problem size to the available resources, or keeping the problem size constant. For multiscale models in this category where *s*_1_ and *s*_2_ execute sequentially, there is no performance benefit, only a large increase in resource consumption. Instead we compare running *s*_1_ and *s*_2_ simultaneously on *a*_1_ (taking times *t*_1_ and *t*_2_), with *s*_1_′ and *s*_2_′ running on *a*_1_ and *a*_2_, respectively. Canals uses strong scaling, implying that *s*_*i*_=*s*_*i*_′, while MultiGrain uses weak scaling, so that *s*_*i*_′ does twice the number of computations as *s*_*i*_. The modified submodels *s*_*i*_′ take time *t*_*i*_′.

For concurrently executing submodels, the local and distributed times are
4.3
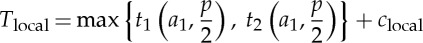

and
4.4
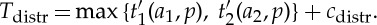



With weak scaling, if *t*_1_(*a*_1_,*p*/2)≈*t*_1_′(*a*_1_,*p*) and *t*_2_(*a*_1_,*p*/2)≈*t*_2_′(*a*_2_,*p*), it is possible to increase the problem size by a factor of 2 without significantly increasing the compute time, as long as the compute time is larger than the communication time. With strong scaling, if *t*_1_(*a*_1_,*p*/2)>*t*_1_′(*a*_1_,*p*) and *t*_2_(*a*_1_,*p*/2)>*t*_2_′(*a*_2_,*p*), and the communication time is not too long, the compute time may decrease.

The results for the applications in this category are shown in table 3. For Canals, a speed-up is not realized for a low-resolution domain size, as the computation time is too short compared with the communication time. For a high resolution, combining the Gordias cluster with the Scylla cluster means computing the same problem 1.4 times faster, consuming 1.4 times more resources. When comparing a distributed run with an equivalent monolithic model, the gain is even larger, with 1.8 times faster calculation. For time-dependent runs where high accuracy is required and local resources are limited, distributed computing turns out to be advantageous for Canals. Previous benchmarks of the Canals application showed small differences between local and distributed calculations of the same problem size with the same number of cores [30], when using MUSCLE. For MultiGrain, it simply means moving from a local desktop to the grid, by being able to use multiple nodes. With the additional computational power, it can search larger parameter spaces in a more stable timeframe, at the expense of consuming more CPU hours.

**Table 3.** Performance measures of scalable multiscale models Canals and MultiGrain. The Canals simulation is performed on the Gordias cluster and the Scylla cluster, with *T*_local_ taken as the average of the *T*_local_ of Gordias and Scylla (only *T*_*local*_ of Gordias between parentheses). It is compared with running the same two submodels at lower core counts (on 50+50 cores) and with running a single monolithic model with the same total problem size (on 100 cores). The time listed for Canals is the time per iteration. The time listed for MultiGrain is the average over 10 simulations and includes the standard error from the mean caused by the stochastic optimization method used. It combines a node of the Zeus cluster and one from the Inula cluster.

simulation	*p*_local_	*T*_local_ (s)	*p*_distr_	*T*_distr_ (s)	speed-up	resources used
Canals (low resolution)	50+50	0.015	100+100	0.023	0.63	3.2
	100	0.011 (0.011)	100+100	0.023	0.47 (0.47)	4.2 (4.3)
Canals (high resolution)	50+50	0.99	100+100	0.71	1.4	1.4
	100	1.77 (1.307)	100+100	0.71	1.8 (2.5)	1.1 (0.80)
MultiGrain	7	27±7	7+4	20±3	1.4	1.1
MultiGrain	11	43±16	11+8	36±10	1.2	1.5

### (c) Skewed multiscale computing

Although the ISR3D and Nano models run on a single large machine without problems, they do not make efficient use of the available CPUs, as some submodels scale very well while others scale hardly at all. There is a large difference between the resource usage of cyclic and acyclic coupling topologies in this case: cyclic coupling topologies involve feedback and thus force resources to be used for the duration of the entire simulation, whereas acyclic coupling topologies do not have feedback so each submodel may be scheduled for exactly the time slot that it needs. Both cluster policies and software would need to be adapted to allow online scheduling of simulations with cyclic coupling topologies, by allowing frequent short reservations, running single iterations of submodels. Without such a change, there will always be some inefficiencies in a simulation due to cumulative queuing time.

The performance model is *A*(*s*_1_)=*A*(*s*_2_)={*a*_1_,*a*_2_}, with *p*_*i*_ resources used on *a*_*i*_, *p*_1_>*p*_2_, *t*_1_(*a*_1_,*p*_1_)≪*t*_1_(*a*_2_,*p*_2_) and *t*_2_(*a*_1_,*p*_1_)≈*t*_2_(*a*_1_,*p*_2_)≈*t*_2_(*a*_2_,*p*_2_). For local sequentially executing submodels, the makespan equation is

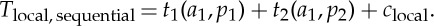

For concurrently executing submodels,

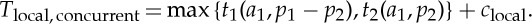

The resources usage becomes *R*_local, mode_=*p*_1_*T*_local, mode_.

For distributed submodels, the makespan equations become
4.5
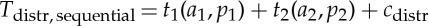

and
4.6
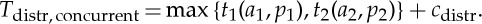



For both the sequential and the concurrent cases, there is no real benefit to makespan with distributed computing, unless submodel 2 computes much faster on another architecture (*t*_2_(*a*_2_,*p*_2_)≪*t*_2_(*a*_1_,*p*_2_)) or if the simulation is slower due to contention between submodels when they run on the same resource (*t*_1_(*a*_1_,*p*_1_)≪*t*_1_(*a*_1_,*p*_1_−*p*_2_)). The negative effects of this may be negligible if the distributed communication time (*c*_*distr*_−*c*_local_) is relatively small. The value may come from lower resource usage, which for the distributed case depends very much on whether the coupling topology is cyclic or acyclic:
4.7
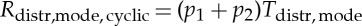

and
4.8
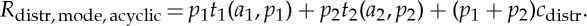



The Nano model [39] has an acyclic coupling topology, and by running each submodel on an appropriate resource with an appropriate number of processors, its resource usage is much less than running all codes in a single reservation. This is primarily because the atomistic calculations, and especially the quantum mechanics calculations, do not run as efficiently on high core counts as the coarse-grained molecular dynamics calculations. In table 4, Nano has a speed-up of 1.7 (equates to multiple days) by going from a single 128 core reservation to combining that with a reservation with 1024 cores. Using multiple distributed reservations instead of one reservation of 1024 or 2048 cores reduces the amount of resources used by five or nine times, respectively.

**Table 4.** Performance measures of skewed multiscale models Nano and ISR3D. The time listed for ISR3D is the time per iteration. The last two rows concern a previous version of ISR3D; it was executed on Huygens and Zeus. The current version was executed on Cartesius and Reef.

simulation	*p*_local_	*T*_local_ (s)	*p*_distr_	*T*_distr_ (s)	speed-up	resources used
Nano	128	9.8×10^5^	64+128+1024	5.7×10^5^	1.73	0.88
Nano	1024	5.7×10^5^	64+128+1024	5.7×10^5^	1.0	0.19
Nano	2048	5.4×10^5^	64+128+2048	5.4×10^5^	1.0	0.11
ISR3D	144	281	144+8	283	0.99	1.06
ISR3D versus alt.			144+8	531	0.53	1.00
ISR3D/old	32	1813	32+4	1532	1.18	0.95
ISR3D/old versus alt.			32+4	1804	1.00	0.56

The two most demanding submodels of ISR3D run sequentially, in a cyclic topology. Thus, simulations would not become more efficient by using distributed computing, were it not for a technique that allows the submodels to run concurrently: running two simulations at once, coordinated so that their submodels alternate their execution. This increases the makespan (originally *T*_local, sequential_) and may decrease the resource usage (originally *R*_local, sequential_), since two simulations are calculated at once. In equations
4.9
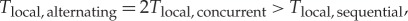

4.10
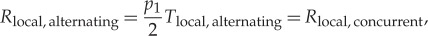

4.11
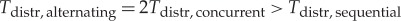

and
4.12
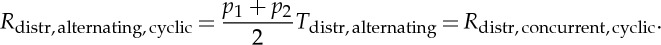



The speed-up stays close to 1, *Sp*=*T*_local,sequential_/*T*_distr,alternating_>1/(1+*ϵ*) for a small *ϵ*, and the resource usage decreases, *U*=*R*_distr,alternating,cyclic_/*R*_local,sequential_<1, if
4.13
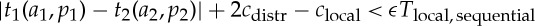

and
4.14
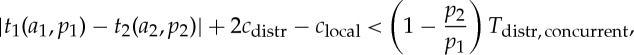

respectively. In words, the increase in makespan is limited and the resource usage is decreased as long as the two submodels take a similar amount of time and the distributed communication time is relatively small.

The benefit in this case is more subtle and presents itself only on certain architectures. As shown in table 4, there was a benefit for ISR3D when a simulation was distributed over Huygens and Zeus [23,24], but not when using Cartesius and Reef (see table 2 for resource details). This was caused by changes in the submodel codes, making them compute one iteration faster and more efficiently, and in the hardware architectures, where a Fortran code would be slower on Huygens than on Zeus due to the compiler and processor type.

### (d) Common benefits

Besides the performance benefits outlined in the previous sections, the applications each benefit from the modularity of MML and the scale separation map [13,15,16]. In particular, MultiGrain, Canals, ISR3D and TTE make active use of the plug-and-play character of MML. The first two do this by changing the coupling topology based on the problem under study, ISR3D and TTE by easily turning on and off certain submodels for validation purposes and by interchanging similar solvers with different numerical properties. For TTE, it is a way to allow combining legacy code into a modern application, whereas HemeLB is able to combine separately developed codes.

A more detailed treatment of the conceptual motivation for this approach can be found in the treatment in this issue by Chopard *et al.* [14].

## 5. Conclusion

The overheads incurred by distributed multiscale computing have been discussed in the literature [19,23]. In this study, we highlight the benefits, which clearly depend on the details of the application. We identified three types of benefits: supplementing local dependencies with HPC resources, increasing the total number of available processors, and load balancing of small- and large-scale resources. Other situations have been excluded from the study and are presumed to favour local execution. For tied multiscale models, the speed-up is highly dependent on the power of the local resources: if the core count is high locally, the speed-up will be less if the local core count is very low, but there will be a speed-up nonetheless. For scalable multiscale models, distributed multiscale computing decreases the computation time while consuming a few more resources if the ratio of computation versus communication is high enough. In practice, this turns out to be at least 1 s of computation for every message sent. For skewed multiscale models, the main advantage of distributed computing is realized in acyclic coupling topologies, where each submodel can easily be distributed with workflow software, choosing appropriate computing resources for each step of the simulation. This final advantage, however, may also be achieved on local resources that allow multistep simulations, partitioned into appropriate reservations. However, starting with a suitable workflow system or runtime environment allows a user to choose either a local or distributed simulation without much further effort.

For skewed applications with cyclic coupling topologies, an advantage is realized only if part of a model computes faster on one resource and the other part on another. It may still benefit from faster compute times by using more (distributed) resources, though, as in the second category. Getting more efficient simulations for cyclic coupling topologies would require a change in the way jobs are scheduled and coupling software is implemented. First of all, advance reservation would have to be used to separately schedule each iteration of a model, possibly using a task graph representation of the execution [13]. Second, a runtime environment would have to start and restart submodels for single iterations, preferably interacting with the model to get the timings of the reservations right. While the second can be implemented in software, the first also requires a policy change for existing clusters and supercomputers. The gain of this approach is that only the resources that are really needed are reserved. Since a separate reservation needs to be made for each iteration, those reservations may as well be made on several, and suitable, resources.

There are many studies on scheduling multisite jobs, particularly in a grid environment, taking into account co-allocation [41], network topology [42] or neither [43–46]. We use manual scheduling in this work to show the applicability to multiscale models but automatic scheduling is essential to make full use of distributed computing. Work that considers concurrent sub-jobs as independent is not directly applicable to cyclic multiscale models, where some submodels are necessarily co-allocated. In practice, researchers have access to a limited number of computing sites, making the question of network topology rather straightforward to evaluate from their point of view. However, if general subscriptions to a range of resources were available, topology-related scheduling decisions become all the more important.

Given the performance models in this work, researchers can make an informed decision on whether to pursue distributed multiscale computing. Whether a benefit is apparent will depend on the infrastructure and the models used.

## Supplementary Material

Performance measurements for the MAPPER applications

## Footnotes

1 MAPPER project website: www.mapper-project.eu.

2 The number of core hours is calculated as the number of cores reserved multiplied by the time for which they are reserved. For example, on a local resource it becomes simply, *R*_local_=*pT*_local_.

## Appendix A. Application and model details

**(a) Tokamak plasma (TTE, fusion)**

The chosen fusion application TTE [47] simulates the time evolution of profiles in the core plasma of a tokamak. This application consists of three submodels: a transport equations solver to evolve the profiles, a fixed-boundary equilibrium code to provide geometry of the plasma, and a turbulence code to calculate anomalous transport coefficients. Different versions of such submodels have been developed within the EDFA Task Force on Integrated Tokamak Modelling (ITM).^3^ The structured data for these submodels are stored in a local database and accessed through a specific library on the Gateway cluster in Garching. The coupled application is implemented using MUSCLE 2.

We have chosen to run this benchmark using only two submodels for the sake of conciseness (the equilibrium is considered fixed during this simulation): the transport solver coming from the European Transport Solver (ETS) [48] and turbulence coming from a gyrofluid approximation flux-tube code GEM [49].

The use case corresponds to a small tokamak plasma (e.g. ASDEX-Upgrade), where ETS evolves temperature and density profiles for electrons and one ion species, and GEM runs on eight flux surfaces, each of which is calculated independently, using time averaging to provide transport coefficients on transport time scale. We have run this case on the Gateway cluster in Germany (GEM up to 256 cores) using fully the database access, and on the Helios supercomputer in Japan (GEM up to 1024 cores) using a tedious ad-hoc setting to simulate the database. With a transport time step at 0.01 s, such application can simulate 10 s of physical time in less than 17 h on 1024 cores (Helios). The model has improved our understanding of the impact of the different submodel parameters on simulation stability at longer time frames (time averaging, equilibrium model limitations, geometric representation), which is one of the main obstacles to overcome before applying such models to predictive simulations on ITER-sized tokamaks. When distributed simulations are allowed on the Helios supercomputer, it will be possible to run such simulations routinely on small to medium tokamak cases in order to validate the approach using experimental data stored in the local database. Finally, the main goal is to run large tokamak cases (ITER will require a grid at least 64 times bigger), to replace the gyrofluid approximation by a gyrokinetic code and use additional submodels (e.g. heating sources) and to complete the description of the physics.

**(b) Cerebrovascular blood flow (HemeLB, biomedicine)**

In this application, we use distributed multiscale computing to connect flow models of the major vessels arterial tree to models of local cerebral vasculature [23]. We do this to improve the accuracy of our simulations of cerebral aneurysms [50] and to understand the effect of patient-specific properties (e.g. heart rate or the structure of the circle of Willis) on the flow dynamics in local cerebral vasculature and aneurysms. Our main motivation to rely on MAPPER is to allow each code to be used in an optimal environment and to minimize the time to completion for the overall system.

The HemeLB model couples two submodels together: PyNS and HemeLB. We use PyNS [51] to model the arterial tree in the human body, and HemeLB to model a local network of arteries in the brain. The two applications exchange pressure values at the boundaries, with the HemeLB simulation domain being a small subset of the overall network modelled in PyNS.

We configured PyNS, which is a zero/one-dimensional discontinuous Galerkin solver, with a 70 bpm heart rate, 90 mmHg mean pressure and  cardiac output. The submodel runs for 4000 time steps on a local desktop at UCL in London (four cores), as it has a number of Python dependencies and does not scale to large core counts. PyNS exchanges pressure values with HemeLB at every time step.

We configured HemeLB, which is a three-dimensional lattice-Boltzmann simulation environment, with a vascular network model with 4.2 million lattice sites, each site sized 3.5×10^−5^ m. It is run for 40 000 time steps on the HECToR supercomputer (512 or 2048 cores), which is located at EPCC in Edinburgh. HemeLB exchanges pressure values with PyNS at every 10 time steps (for a total of 4000 exchanges).

The codes are coupled with the MPWide communication library.

**(c) Irrigation network (Canals, hydrology)**

The water service demands are increasing rapidly. They are driven by several factors like the impact of climatic conditions, agriculture, overpopulation, etc. [52]. Today, the technical heritage to manage water resources and complex irrigation systems has become somewhat insufficient to cope with the new urgent needs and to predict in advance solutions for emergency cases (e.g. natural hazards). One way to deal with this is to use numerical models and computer simulations as a decision-making tool.

One of our interests in collaboration with the Geneva electricity company is to simulate a section of the Rhone river (13 km), from Geneva city down to Verbois water dam [29]. This simulation investigates the possibility to study specific questions, namely the way to flush sediment in some critical areas by changing the water levels. This requires a three-dimensional free surface (3DFS) model with sediment to study these critical areas and one-dimensional shallow water to study areas where the flow behaviour is stable. The 3DFS model requires supercomputer capabilities contrary to the one-dimensional model, which can be executed on clusters or desktop machines. Coupling 3DFS and one-dimensional models results in a multiscale model and simulation [19]. In addition, using 3DFS with high resolution to get more accurate results cannot be afforded by what a local centre can provide in terms of computing power. The MAPPER framework allows one to easily connect these different components and run them on a distributed grid of supercomputers [33].

**(d) Reverse engineering of gene regulatory networks (MultiGrain, systems biology)**

Gene regulation networks (GRNs) can be viewed as the ‘cognitive architecture’ in living cells; they control how much product (protein, RNA) is synthesized and when in response to input from the external or internal environment. Modelling and simulation of GRNs is an unsolved problem [53]. Furthermore, future models of GRNs are likely to be considerably more complex (number of genes, biochemical and physical levels involved) than their current counterparts. Our vision is to enable modelling and simulation of large gene regulation networks that exhibit an inherent subdivision particularly in the time dimension. Such gene networks typically consist of dozens or hundreds of genes. They are difficult to handle with conventional modelling and simulation approaches due to the conceptual and computational complexities involved.

MultiGrain/MAPPER is a distributed multiscale computing application designed for modelling and simulation of large GRN systems based on MAPPER multiscale computing solutions. MultiGrain is a Java library providing a number of GRN reverse engineering features, including flexible specification of GRN rate laws, flexible algorithms separating GRN structure inference and time-course data fitting, and distributed optimization based on a particle swarm optimization [54] approach enabled by MAPPER components including MUSCLE 2 and QosCosGrid. Consequently, MultiGrain is able to run on a variety of hardware from personal computers to multi-processor architectures, computer clusters or even supercomputers.

**(e) Clay–polymer nanocomposites (Nano, nanomaterials)**

In this application, we assess the material properties of clay–polymer nanocomposites. The nanocomposites fall within the realm of the emergent area known as nanotechnology, where materials are designed and built at the atomic level, an area of science currently at the forefront of academic, industrial, health and public interest. Tailoring the clay structure on the nanometre scale produces composites with novel material properties, which can be significantly different from bulk properties and have already been applied in numerous commercial applications, such as in the automotive industry, biodegradable food packaging and in oil drilling fluids.

Our multiscale model involves four stages of execution:

(i) Parametrizing the potentials of clay sheet edges with CPMD [55], using up to 64 cores on a local cluster (Mavrino at University College London).
(ii) Modelling basic clay sheets and polymers using LAMMPS [56] in all-atom mode (the former with the previously calculated potentials), using up to approximately 1024 cores on HECToR.
(iii) Iteratively coarse-graining and optimizing the potentials using the iterative Boltzmann inversion (a few of the potentials were done using force matching), to construct accurate coarse-grained potentials, using about 32 cores on Mavrino or HECToR.
(iii) Executing coarse-grained production simulations using these newly found potentials, using thousands or tens of thousands of cores on HECToR.

In this paper, we presented the performance of our simulations for one particular production system, which contains montmorillonite clay and uncharged polyvinyl alcohol.

**(f) In-stent restenosis (ISR3D, biomedicine)**

A common expression of coronary heart disease is arteriosclerosis: thickened and hardened blood vessels caused by a build-up of atheromatous plaque. This is associated with a partial occlusion of the blood vessel lumen, called a stenosis. A common intervention to open up the lumen is balloon angioplasty, assisted by a stent that keeps the lumen open after the intervention. An adverse response to the stent, a regrowth of tissue, may result in a re-occlusion of the lumen, called in-stent restenosis [57,58]. The exact causes and key factors of in-stent restenosis are not yet known [59]. The three-dimensional model of in-stent restenosis ISR3D models the response to the stent-assisted angioplasty, taking into account tissue damage due to the stent placement, smooth muscle cell proliferation and migration, shear stress caused by blood flow and the effect of local drug diffusion if a drug-eluting stent [60–62].

ISR3D has a cyclic coupling topology, alternating mainly between a smooth muscle cell submodel and a blood flow submodel. The smooth muscle cell code is parallelized with OpenMP, limiting it to a single compute node, while the blood flow code uses a lattice Boltzmann library, Palabos,^4^ that has MPI parallelization and scales well [24]. Depending on the blood vessel geometry and the resolution of the blood flow solver, a simulation consisting of 1500 time steps takes a few days to more than a week.
